# Quality of life of depressed patients with chronic diseases

**DOI:** 10.1192/j.eurpsy.2023.1772

**Published:** 2023-07-19

**Authors:** M. G. Rojas, V. Guajardo, P. Martinez

**Affiliations:** Psychiatry and mental helath, clnical hospital, Santiago, Chile

## Abstract

**Introduction:**

Chronic diseases are a public health problem and high prevalent on depressed patients.

**Objectives:**

To describe the sociodemographic characteristics and the quality of life of a sample of depressed patients with hypertension and oder diabetes as comorbidity.

**Methods:**

It is the baseline evaluation of 361 persons participating in a clinical trial that studies the effectiveness of a psychoeducational intervention for this type of patients.

Persons with moderate or severe depression and with hypertension and or diabetes attending 8 primary care centers in Santiago were invited to participate .

**Results:**

The sample consisted of 361 study participants,the majority female(89.97%).The mean age was 59.81 years(de=10.28),with an age range observed from 26 to 83 years.Most of the participants had primary(35,91%)or secondary (43.21%)education level.More than a third of the participants reported houshold chores(34.09%) and a quarter were working for income(28.41%).About half of the participants were married(44.48%).The mean PHQ-9 score was 18.73(sd=2.81).Most of the participants had a previous diagnoses of depression(60.39%).The sample obtained an average of 34.99 points(sd=20.82) in the mental health component of the 12-item Short Form Health Survey indicative of poor quality of life related to mental health.

**Conclusions:**

Depressed patients with chronic diseases ,users of primary care clinics, have poor quality of life,so it is urgent to review care protocols to achieve better health results.

**Disclosure of Interest:**

None Declared

